# Taxane combined with lobaplatin or anthracycline for neoadjuvant chemotherapy of triple-negative breast cancer: a randomized, controlled, phase II study

**DOI:** 10.1186/s12916-024-03474-0

**Published:** 2024-06-18

**Authors:** Cheng Wang, Long Yuan, Xiujuan Wu, Yan Wang, Hao Tian, Guozhi Zhang, Andi Wan, Siyi Xiong, Chengfang Wang, Yuqin Zhou, Dandan Ma, Yangqiu Bao, Man Qu, Jun Jiang, Yi Zhang, Xiaowei Qi

**Affiliations:** Department of Breast and Thyroid Surgery, Southwest Hospital, Army Medical University, Chongqing, China

**Keywords:** Neoadjuvant chemotherapy, TNBC, taxane, Lobaplatin, Anthracycline, pCR

## Abstract

**Background:**

Previous studies have shown that the addition of platinum to neoadjuvant chemotherapy (NAC) improved outcomes for patients with triple-negative breast cancer (TNBC). However, no studies have assessed the efficacy and safety of the combination of taxane and lobaplatin. In this study, we conducted a randomized controlled phase II clinical study to compare the efficacy and safety of taxane combined with lobaplatin or anthracycline.

**Methods:**

We randomly allocated patients with stage I–III TNBC into Arm A and Arm B. Arm A received six cycles of taxane combined with lobaplatin (TL). Arm B received six cycles of taxane combined with anthracycline and cyclophosphamide (TEC) or eight cycles of anthracycline combined with cyclophosphamide and sequential use of taxane (EC-T). Both Arms underwent surgery after NAC. The primary endpoint was the pathologic complete response (pCR). Secondary endpoints were event-free survival (EFS), overall survival (OS), and safety.

**Results:**

A total of 103 patients (51 in Arm A and 52 in Arm B) were assessed. The pCR rate of Arm A was significantly higher than that of Arm B (41.2% *vs*. 21.2%, *P* = 0.028). Patients with positive lymph nodes and low neutrophil-to-lymphocyte ratio (NLR) benefited significantly more from Arm A than those with negative lymph nodes and high NLR (*P*_interaction_ = 0.001, *P*_interaction_ = 0.012, respectively). There was no significant difference in EFS (*P* = 0.895) or OS (*P* = 0.633) between the two arms. The prevalence of grade-3/4 anemia was higher in Arm A (*P* = 0.015), and the prevalence of grade-3/4 neutropenia was higher in Arm B (*P* = 0.044).

**Conclusions:**

Neoadjuvant taxane plus lobaplatin has shown better efficacy than taxane plus anthracycline, and both regimens have similar toxicity profiles. This trial may provide a reference for a better combination strategy of immunotherapy in NAC for TNBC in the future.

**Supplementary Information:**

The online version contains supplementary material available at 10.1186/s12916-024-03474-0.

## Background

Breast cancer has become the most common cancer worldwide and one of the leading causes of cancer-related death based on global cancer burden estimates for 2020 [1]. By the year 2070, 4.4 million new cases of breast cancer are expected to be diagnosed worldwide [2]. About 15–20% of all cases of breast cancer are considered triple-negative breast cancer (TNBC), a subtype of breast cancer with poor prognosis [3]. Approximately 16% of patients with TNBC experience local or distant recurrences within five years, and the median overall survival for metastatic TNBC is only 13.5 months [4, 5].

For the last few years, platinum-based drugs like cisplatin, carboplatin, and lobaplatin have been widely used for TNBC in neoadjuvant chemotherapy (NAC) settings, increasing the pCR rate and improving disease-free survival (DFS) and overall survival (OS) [6]. Many researchers have stated that most NAC regimens for TNBC should include platinum [7]. Lobaplatin is a platinum-based third-generation anticancer agent approved in China for the treatment of advanced breast cancer, small-cell lung cancer, and chronic myeloid leukemia. As we previously reported in a phase-II clinical trial and 5-year follow-up study, the addition of lobaplatin to taxane and anthracycline was found to significantly improve overall pathologic and objective response rates and DFS. [8, 9]. A phase II study compared the efficacy and toxicity of TL (docetaxel + lobaplatin) induction chemotherapy combined with lobaplatin concurrent chemoradiotherapy and TPF (docetaxel + cisplatin + 5-fluorouracil) induction chemotherapy combined with cisplatin concurrent chemoradiotherapy in the treatment of locally advanced head and neck squamous cell carcinoma, and the results indicated that patients survived longer and experienced lower toxicity when treated with TL regimen [10]. It has also been demonstrated in a multicenter study of esophageal cancer and a phase III trial of nasopharyngeal cancer that lobaplatin has similar therapeutic effects as cisplatin while presenting lower toxicity [11, 12]. In a study of metastatic breast cancer, lobaplatin had low toxicity and improved therapeutic efficacy over cisplatin [13]. Thus, lobaplatin seems to offer superior prospects for treating breast cancer compared to other platinum-based treatments.

Anthracyclines, as classical chemotherapy agents for breast cancer, have been demonstrated to cause heart toxicity through various mechanisms, and this toxicity can be long-term and ultimately cause heart failure [14, 15]. According to one study, language memory ability was significantly lower in patients treated with anthracyclines than those treated with anthracycline-free chemotherapy [16]. Therefore, the toxicity of anthracyclines cannot be ignored. Anthracycline-containing regimens and anthracycline-free regimens are similar in terms of curative effects for early-stage human epidermal growth factor receptor 2 (HER2)-positive breast cancer and initial treatment of metastatic breast cancer [17–19]. However, no comparison has been conducted between lobaplatin and anthracycline in the treatment of TNBC.

Herein, in this study, we compared the efficacy and safety of taxane combined with lobaplatin versus taxane combined with anthracycline. This study aims to provide evidence-based data to enable a more effective NAC regimen for TNBC.

## Methods

### Study design

This study was an open-label, single-center, randomized, controlled, phase II clinical study. All the participants were Asian women. Arm A was the intervention group and Arm B was the control group. The primary endpoint was pCR. Based on previous literature reports, pCR rate in the control group was estimated to be 21%, while pCR rate in the intervention group was estimated to be 46%. Setting α = 0.05 and 1-β = 0.80, the sample size for Arms A and B was calculated to be 50 using PASS 11. Based on the assumption that 20% of the study population would drop out, a sample size of 60 would be required for both arms. Written informed consent (and a statement confirming consent for publication) was obtained from patients before assigning them to a treatment group. This study was conducted following the principles of the Helsinki Declaration, and results were reported using the Consolidated Standards of Reporting Trials guidelines [20]. This study was approved by the Ethics Committee of the First Affiliated Hospital of Army Medical University. It was registered in the China Clinical Trial Registry (registration number: chictr1900023776, linker: https://www.chictr.org.cn/showproj.html?proj=39908) on 11 June 2019.

### Participants

The inclusion criteria were: (1) age > 18 years; (2) Eastern Cooperative Oncology Group score of 0 or 1; (3) pathologically confirmed clinical stage I–III TNBC; (4) Following NAC, patients underwent appropriate surgical treatment in our hospital (the specific surgical method was determined according to the patient’s response following NAC). The exclusion criteria were: (1) receipt of any type of treatment before NAC; (2) bilateral breast cancer, inflammatory breast cancer, or with a history of other cancers; (3) acute/chronic inflammatory diseases, autoimmune diseases, mental disorders, severe liver/kidney dysfunction, or severe complications. Pathological diagnosis was obtained by core needle biopsy before starting NAC. Immunohistochemical analysis was conducted to evaluate the status of the estrogen receptor (ER), progesterone receptor (PR), and HER2. Triple negativity was defined as ER and PR nuclear staining of ≤ 1%. HER2 negativity was defined as an Immunohistochemistry(IHC) score of 0, 1 + , or 2 + (Fluorescence in situ hybridization not amplified).

### Study procedures

Patients were randomly allocated into Arm A and Arm B (1:1). The randomization sequence was generated with Research Randomizer (www.randomizer.org). The chemotherapy regimen of Arm A was TL (six-cycle docetaxel 75 mg/m2 or albumin-bound paclitaxel 125 mg/m2 + lobaplatin 30 mg/m2). Arm B had two chemotherapy regimens, EC-T (four-cycle anthracycline 90 mg/m^2^ + cyclophosphamide (C) 600 mg/m^2^ followed by four-cycle docetaxel 80 mg/m^2^ or albumin-bound paclitaxel 125 mg/m^2^) and TEC (six-cycle docetaxel 75 mg/m^2^ or albumin-bound paclitaxel 125 mg/m^2^ + epirubicin 75 mg/m^2^ + cyclophosphamide 500 mg/m^2^). In Arm B, the physician selected NAC regimens (TEC or EC-T) based on the patient’s disease status and willingness. Each regimen was administered intravenously on the first day of a three-week cycle.

The Ki-67 index was expressed as the percentage of positive cell counts in ≥ 100 tumor cells. Expression of cytokeratin (ck)5/6 was considered to be negative if nuclear staining was < 1%, and positive if nuclear staining was ≥ 1%. A sample of peripheral blood was collected from patients before their first chemotherapy session. Following the measurement of platelets, neutrophils, and lymphocytes, we calculated the platelet-to-lymphocyte ratio (PLR) and low neutrophil-to-lymphocyte ratio (NLR), and divided them into high/low groups based on the median value. The number of stromal tumor-infiltrating lymphocytes (sTILs) was counted according to recommendations of the 2014 International Working Group on Tumor-Infiltrating Lymphocytes in Breast Cancer [21]. Tumor size and the status of axillary lymph nodes were evaluated by ultrasound within 1 week before NAC and before surgery. For those with negative lymph nodes (before NAC), a sentinel lymph node biopsy was performed, while for those with positive lymph nodes (before NAC), an axillary lymph node dissection was performed.

### Study outcomes

Endpoints were defined according to Standardized Definitions for Efficacy End Points in Neoadjuvant Breast Cancer Clinical Trials: NeoSTEEP [22]. The primary endpoint of our study was the pathologic complete response (pCR). pCR (ypT0/Tis ypN0) was defined as the absence of residual invasive cancer on hematoxylin and eosin evaluation of the complete resected breast specimen and all sampled regional lymph nodes following the completion of NAC. The secondary endpoints were event-free survival (EFS), overall survival (OS), and safety. EFS was defined as the time from randomization to the occurrence of any of the following events: local–regional progression or distant progression before surgery, invasive ipsilateral breast tumor recurrence, local/regional invasive recurrence, distant recurrence and death from any cause. OS was defined as the time from enrollment to death from breast cancer, non-breast cancer, or unknown cause. The tumor response to NAC was measured using Response Evaluation Criteria in Solid Tumors (RECIST) 1.1. Treatment-related adverse events were documented. Toxicity was graded using the general toxicity criteria (version 5.0) set by the US National Cancer Institute. Each patient was scheduled for follow-up visit every 3 months during years 1 to 2, every 6 months for the third to the fifth years, and annually thereafter. A drop-out rate of 10% was allowed. Each follow-up visit included abdominal and breast ultrasound. Mammography and chest radiography were performed yearly. Computed tomography, magnetic resonance imaging, and bone scan were necessary for patients with suspected metastatic lesions.

### Statistical analyses

Data were analyzed by SPSS 26.0 (IBM, Armonk, NY, USA). Differences in baseline characteristics between Arm A and Arm B as well as EC-T group and TEC group of Arm B, and pCR rate as well as treatment-related toxicities between Arm A and Arm B, were compared using Pearson χ2 test, and Fisher Exact Test was used when the minimum expected count was less than 5. The interaction test was done to determine if treatment effects differ between different subgroups of Arm A and Arm B. Kaplan–Meier curves were drawn by R 4.2.2 (R Institute for Statistical Computing, Vienna, Austria), and *P* value was calculated by Log − rank test to evaluate survival differences between Arm A and Arm B as well as between pCR and non-pCR groups. *P* < 0.05 (two-sided) was considered significant.

## Results

### Clinicopathologic features

**Fig. 1:**
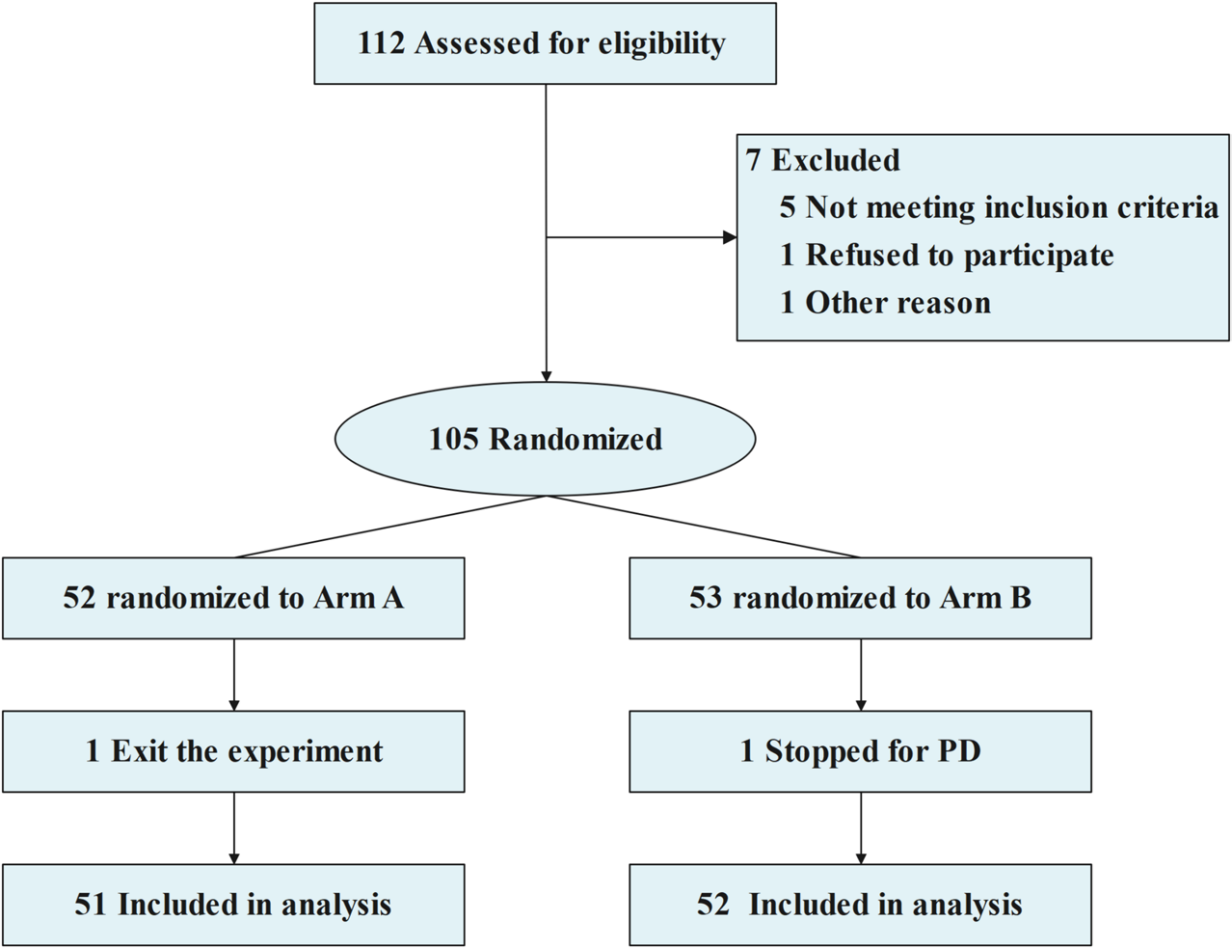
Flowchart showing patient screening

Patients were screened strictly according to the inclusion and exclusion criteria. A total of 112 patients were initially included in the study. Seven patients were excluded according to the exclusion criteria. A total of 105 patients were randomly allocated into Arm A and Arm B. One patient in Arm A withdrew from the clinical trial, and one patient in Arm B stopped treatment due to disease progression. The screening process is shown in Fig. 1. Finally, 103 patients were evaluated: 51 patients in Arm A and 52 patients in Arm B. In Arm B, 25 patients received eight cycles of EC-T therapy, and 27 patients received six cycles of TEC therapy.

Data from both arms were collected and analyzed. The median age of Arm A and Arm B was 49 years and 50 years, respectively. Women in China are most likely to develop breast cancer at the age of 50 [23]. The clinicopathologic characteristics of the two Arms at baseline were comparable. There was no significant difference in age, menstrual status, T stage, lymph node status, Ck5/6 expression, Ki-67 index, HER2 status, or sTILs expression. Notably, compared to Arm A, there were 11.1% more stage III patients in Arm B (34.6% vs. 23.5%), although the difference was not statistically significant (*P* = 0.216). The clinicopathologic characteristics of all patients are presented in Table 1. Because Arm B consists of two chemotherapy regimens, we also examined the baseline characteristics of the EC-T group and TEC group, whereas there was no significant difference in baseline characteristics between both groups. (Additional file 1: Table S1).

**Table 1:** Characteristics of study participants at baseline

Characteristics	Arm A (*N* = 51)	Arm B(*N* = 52)	*P* value^a^
Age, median(range), y	49(29–73)	50(31–70)	0.916^b^
Menopausal status, No.(%)			0.477
Premenopausal	30(60.8)	27(53.8)	
Postmenopausal	21(39.2)	25(46.2)	
T stage, No.(%)			0.741^c^
T1-T2	47(92.2)	46(88.5)	
T3-T4	4(7.8)	6(11.5)	
Lymph node status, No.(%)			0.619
Negative	16(31.4)	14(26.9)	
Positive	35(68.6)	38(73.1)	
Clinical stage, No.(%)			0.216
Stage I-II	39(76.5)	34(65.4)	
Stage III	12(23.5)	18(34.6)	
CK5/6, No.(%)			0.895
Negative	18(35.3)	19(36.5)	
Positive	33(64.7)	33(63.5)	
Ki67, No.(%)			0.616
< 30%	7(13.7)	9(17.3)	
≥ 30%	44(86.3)	43(82.7)	
HER2, No.(%)			0.226
Negative	38(74.5)	33(63.5)	
1 + /2 + (fish-)	13(25.5)	19(36.5)	
sTILs, No.(%)			0.813
< 50%	40(78.4)	39(75.0)	
≥ 50%	11(21.6)	13(25.0)	

^a^Pearson χ2 test

^b^T test

^c^Fisher exact test

### Treatment outcomes

**Fig. 2:**
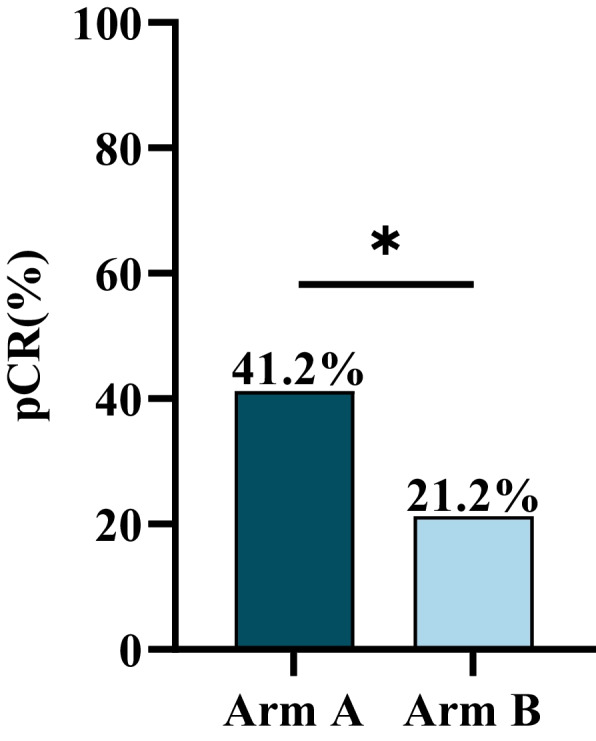
Comparison of pCR rates for Arm A and Arm B. χ^2^ = 4.820, ^*^P < 0.05

Arm A showed a pCR rate of 41.2% (21/51), while Arm B showed a pCR rate of 21.2% (11/52). Arm A had a significantly higher pCR rate (*P* = 0.028) (Fig. 2). The pCR rate for the EC-T group and the TEC group was 20.0% (5/25) and 22.2% (6/27), respectively. (*P* = 0.845). (Additional file 2: Fig. S1).

**Fig. 3:**
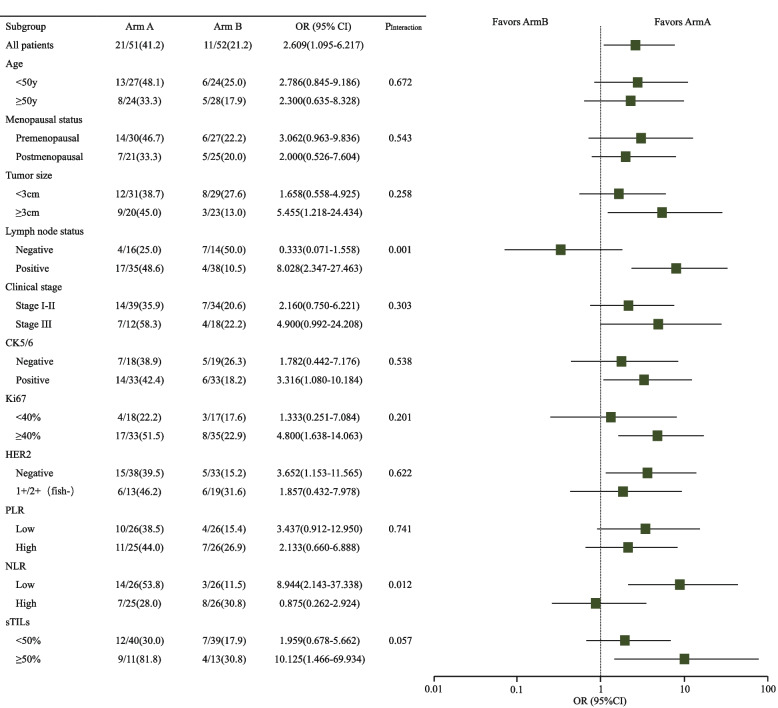
Subgroup analysis shown as a forest plot. Differences between subgroups for Arm A *versus* Arm B

Post hoc exploratory subgroup analyses for pathological complete response are shown in Fig. 3. The patients with positive lymph nodes and low NLR benefited significantly more from Arm A than those with negative lymph nodes and high NLR (48.6% vs 25.0%, *P*_interaction_ = 0.001; 53.8% vs 28.0%, *P*_interaction_ = 0.012, respectively).

**Fig. 4:**
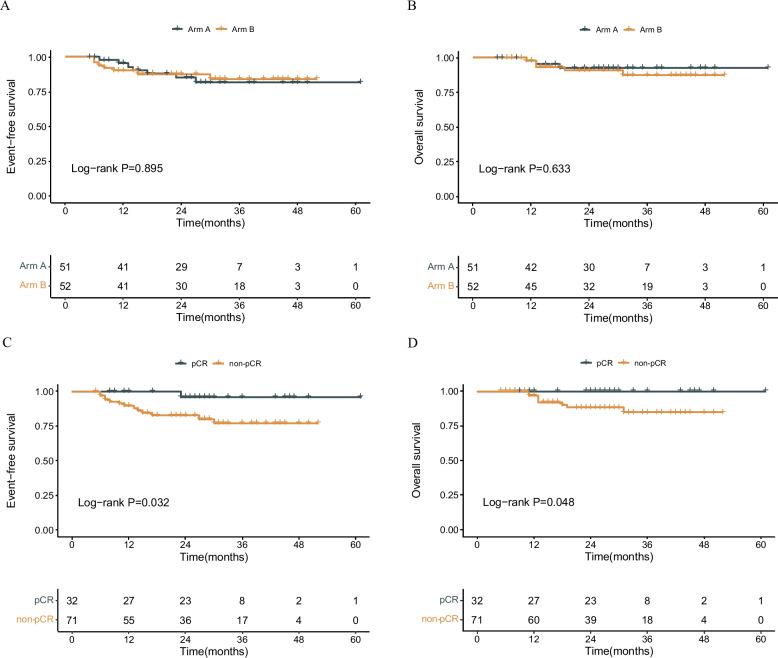
Survival analysis. EFS (A) and OS (B) were compared between Arm A and Arm B. EFS (C) and OS (D) were compared between pCR group and non-pCR group

The median follow-up was 27 months (range, 5–61 months). There were no obvious differences between two arms as for EFS and OS, though the data is still immature (Fig. 4A-B). All patients were divided into two groups based on whether they had achieved pCR or not. As a result, 32 patients were categorized into the pCR group and 71 patients into the non-pCR group. The EFS (hazard ratio [HR] = 0.146, 95%CI = 0.893–52.224, *P* = 0.032) and OS (*P* = 0.048) in the pCR group were significantly longer than in the non-pCR group (Fig. 4C-D). Additionally, we separately analyzed the survival difference between the pCR and non-pCR groups in both Arms A and B. However, no significant difference was found in EFS or OS, though the data is still immature. (Additional file 2: Fig. S2).

### Treatment-related toxicity

Grade 3/4 neutropenia was more prevalent in Arm B than in Arm A (*P* = 0.044), whereas anemia of grade 3/4 was more prevalent in Arm A (*P* = 0.015). (Table 2). There was no significant difference in the prevalence of leukopenia (*P* = 0.291) or thrombocytopenia. In addition, there was no significant difference between the two groups in terms of non-hematological toxicity, including grades 3/4 liver injury, vomiting, diarrhea, phlebitis, subcutaneous hemorrhage, and neuritis (Table 2).

**Table 2:** Grade-3/4 treatment-related toxicity

	Arm A (*N* = 51)	Arm B (*N* = 52)	*P* value^c^
Leukopenia	1	4	0.175
Neutropenia	1	6	0.034
Anemia	11	2	0.015^a^
Thrombocytopenia	2	0	-^d^
Increase in ALT/AST levels	2	3	1.000
Vomiting	0	2	-^d^
Diarrhea	1	0	-^d^
Phlebitis	0	1	-^d^
Subcutaneous hemorrhage	0	0	^−d^
Neuritis	0	1	-^d^

^a^Pearson χ2 test

^c^Fisher exact test

^d^No test

## Discussion

This is the first clinical trial to compare the efficacy and safety of lobaplatin (a third-generation platinum-based drug) with those of classical anthracyclines in the NAC of TNBC. This trial demonstrated that the pCR rate was higher with the combination of taxane and lobaplatin compared to the combination of taxane and anthracycline, and there was no clinically significant difference in toxicity.

Taxanes combined with platinum-based agents have been applied gradually as an alternative adjuvant treatment for TNBC and have also shown promising outcomes, demonstrating that platinum can be effective in treating early-stage TNBC [24–26]. It has been demonstrated in a meta-analysis that platinum-based therapy significantly improves the DFS of patients with early TNBC [27].

There have been several studies comparing carboplatin and anthracyclines as NAC for TNBC. Results from NeoSTOP study revealed no significant difference in the pCR rate, EFS, or OS between regimens of sequential use of doxorubicin and cyclophosphamide (TCb-EC) and taxane combined with carboplatin (TCb). However, grade 3 or 4 adverse events were more common in the TCb-EC group than in the TCb group [28]. NeoCART study also showed that compared with a regimen comprising eight-cycle anthracycline combined with cyclophosphamide and sequential use of taxane (EC-T), the regimen of taxane combined with carboplatin (TCb) could improve the pCR rate of TNBC further [29]. According to these data, the cessation of anthracycline use in a platinum-containing regimen may achieve a similar therapeutic effect.

In a study of 120 cases of ovarian cancer, patients in the lobaplatin group had significantly lower levels of HE4 and CA125 than those in the carboplatin group when examined at 3 or 6 months after chemotherapy (*P* < 0.05) [30]. A study of 68 cases of advanced inoperable esophageal cancer reported the treatment response rates was 73.53% and 50.00% in the TL (paclitaxel/lobaplatin) and PF (cisplatin/5-fluorouracil) treatment groups, respectively (*P* = 0.040). The median progression-free survival was 13.0 and 6.5 months in the TL and PF groups, respectively (*P* = 0.034) [31]. In a study of 87 cases of metastatic breast cancer, lobaplatin-based regimen was found to improve progression-free survival (PFS) when compared to cisplatin-based regimen (median 13.2 vs 4.7 months, HR = 0.37, 95% confidence intervals: 0.21–0.67, *P* = 0.0007) [32]. According to these studies, lobaplatin may be more effective than other platinum-based agents.

There were significant interaction effects between NLR as well as lymph node status and chemotherapy regimens. NLR is well known as a prognostic marker for cancers, such as breast cancer, urological cancer, and head and neck cancer, and patients with low NLR have a better prognosis than those with high NLR [13, 33, 34]. In addition, a study of 61 TNBC patients receiving the EFC-T (fluorouracil, epirubicin, and cyclophosphamide sequential paclitaxel) neoadjuvant regimen found that patients with low NLR had a higher pCR rate than those with high NLR (72.2% vs. 8.0% *P* < 0.001) [35]. Similarly, another study involving 87 cases of TNBC treated with taxane and anthracycline neoadjuvant therapy found that patients with low NLR achieved a higher pCR rate than those with high NLR. (42.1% vs. 18.4%, *P* = 0.018) [36]. In addition, our previous study found that patients with low NLR are more likely to benefit from lobaplatin-containing NAC regimens, with pCR rates of 49.1% and 23.3% in the low and high NLR groups, respectively (*P* = 0.024) [37]. It could be noted that high NLR is linked to inflammation and high NLR promotes tumor development through the inflammatory microenvironment [38, 39]. Therefore, patients with low NLR may benefit more from neoadjuvant chemotherapy. Besides that, patients with positive lymph nodes benefited significantly more from the lobaplatin regimen (Arm A) than those with negative lymph nodes. This result differs from BrighTNess and NeoCART primarily due to the relatively small sample size studied and/or the different therapeutic effects between lobaplatin and carboplatin/cisplatin.

A study about TNBC showed that node-positive patients had lower pCR rates than node-negative patients (22.0% vs 48.7%, *P* = 0.006) [40]. According to the results of the current study, our control group had a higher proportion of node-positive patients (68.6%) than other studies, such as CALGB 40603 (58%) [41], BrighTNess (43%) [42], and KEYNOTE-522 (44.1%) [43], which might result in lower pCR rate in our control group. Nonetheless, the pCR rate fell within the reported range for patients with TNBC who received NAC, according to a meta-analysis (14%-53.4%) [44].

Many studies have demonstrated the importance of pCR in evaluating the efficacy of NAC and patients who achieve a pCR tend to have a better prognosis as well as a longer life expectancy [45–47]. I-SPY 2, a neoadjuvant adaptive trial and platform, was developed to improve outcomes in high-risk breast cancer. It has redefined response-based subtypes and treatment priorities for breast cancer treatment [48]. As demonstrated in I-SPY2, effective neoadjuvant treatment altered residual cancer burden distribution, increased pCR, and improved EFS for breast cancer patients [49, 50]. This is expected to be the case in our current practice as well, although the current data is immature, and long-term follow-up is needed. As for toxicity, in combination with the results of the current study and two previous studies conducted by our team on lobaplatin, we hypothesize that, compared to the regimen of taxane plus anthracycline, the TL regimen increases the risk of grade-3/4 anemia, but decreases the risk of grade-3/4 neutropenia [8, 9].

There are some limitations to this clinical trial. As this was a single-center clinical trial with a small sample size, there may be varying degrees of bias in the data. Long-term outcomes of the two regimens remain to be determined due to short-term follow-up and fewer secondary endpoint events. Since we initiated this trial prior to the publication of the KEYNOTE-522 study, immunotherapy was not included in this trial, although immunotherapy has now become a crucial part of neoadjuvant treatment for TNBC. National Comprehensive Cancer Network guidelines recommend that patients with TNBC be treated with a dose-dense regimen. However, many patients in Chinese are not suitable for this regimen due to tolerance issues and other factors, which is why the dose-dense regimen was not selected for this study. Considering the small sample size and limited number of biomarkers included in this study, the results of the subgroup analysis should be interpreted with caution.

## Conclusions

Neoadjuvant taxane plus lobaplatin has shown better efficacy than taxane plus anthracycline, and both regimens have similar toxicity profiles. This trial may provide a reference for a better combination strategy of immunotherapy in NAC for TNBC in the future.

### Supplementary Information

Additional file 1: Table S1. Characteristics of EC-T and TEC group participants at baseline.

Additional file 2: Figures S1-S2. Fig. S1- [Comparison of pCR rates for EC-T and TEC group]. Fig. S2 –[Comparison of  EFS  and OS  between pCR group with non-pCR group for Arm A and B].

## Data Availability

The datasets generated during the current study are available from the corresponding author on reasonable request.

## Abbreviations

EFS: Event-free survival
ER: Estrogen receptor
HER2: Human epidermal growth factor receptor 2
IHC: Immunohistochemistry
NAC: Neoadjuvant chemotherapy
NLR: Neutrophil-to-lymphocyte ratio
OS: Overall survival
pCR: Pathologic complete response
PLR: Platelet-to-lymphocyte ratio
PR: Progesterone receptor
sTILs: Stromal tumor-infiltrating lymphocytes
TNBC: Triple-negative breast cancer
