# Supplementary material for: Taxane combined with lobaplatin or anthracycline for neoadjuvant chemotherapy of triple-negative breast cancer: a randomized, controlled, phase II study
Source: BMC Med. 2024 Jun 18;22:252. doi: 10.1186/s12916-024-03474-0 (PMC11184884; doi:10.1186/s12916-024-03474-0)
Supplement: Supplementary file 1 — Additional file 1: Table S1. Characteristics of EC-T and TEC group participants at baseline. [file 12916_2024_3474_MOESM1_ESM.docx]

Additional File 2: Table S1. Characteristics of EC-T and TEC group participants at baseline

| Characteristics | EC-T (N=25) | TEC(N=27) | *P* value^a^ |
| --- | --- | --- | --- |
| Age, median(rang), y | 48(32-70) | 52(31-65) | 0.331^b^ |
| Menopausal status, No.(%) |  |  | 0.093 |
| Premenopausal | 16(64.0) | 11(40.7) |  |
| Postmenopausal | 9(36.0) | 16(59.3) |  |
| T stage, No.(%) |  |  | 0.411^c^ |
| T1-T2 | 21(84.0) | 25(92.6) |  |
| T3-T4 | 4(16.0) | 2(7.4) |  |
| Lymph node status, No.(%) |  |  | 0.647 |
| Negative | 6(24.0) | 8(29.3) |  |
| Positive | 19(76.0) | 19(70.7) |  |
| Clinical stage, No.(%) |  |  | 0.840 |
| Stage I-II | 16(64.0) | 18(66.7) |  |
| Stage III | 9(36.0) | 9(33.3) |  |
| CK5/6, No.(%) |  |  | 0.938 |
| Negative | 9(36.0) | 10(37.0) |  |
| Positive | 16(64.0) | 17(63.0) |  |
| Ki67, No.(%) |  |  | 1.000^c^ |
| <30% | 4(16.0) | 5(18.5) |  |
| ≥30% | 21(84.0) | 22(81.5) |  |
| HER2, No.(%) |  |  | 0.710 |
| Negative | 19(76.0) | 14(51.9) |  |
| 1+/2+（fish-） | 6(24.0) | 13(48.1) |  |
| sTILs, No.(%) |  |  | 0.078 |
| <50% | 16(64.0) | 23(85.2) |  |
| ≥50% | 9(36.0) | 4(14.8) |  |

^a^Pearson χ2 test, ^b^T test, ^c^Fisher exact test.
