# Supplementary material for: Taxane combined with lobaplatin or anthracycline for neoadjuvant chemotherapy of triple-negative breast cancer: a randomized, controlled, phase II study
Source: BMC Med. 2024 Jun 18;22:252. doi: 10.1186/s12916-024-03474-0 (PMC11184884; doi:10.1186/s12916-024-03474-0)
Supplement: Supplementary file 2 — Additional file 2: Figures S1-S2. Fig. S1- [Comparison of pCR rates for EC-T and TEC group]. Fig. S2 –[Comparison of EFS and OS between pCR group with non-pCR group for Arm A and B]. [file 12916_2024_3474_MOESM2_ESM.docx]

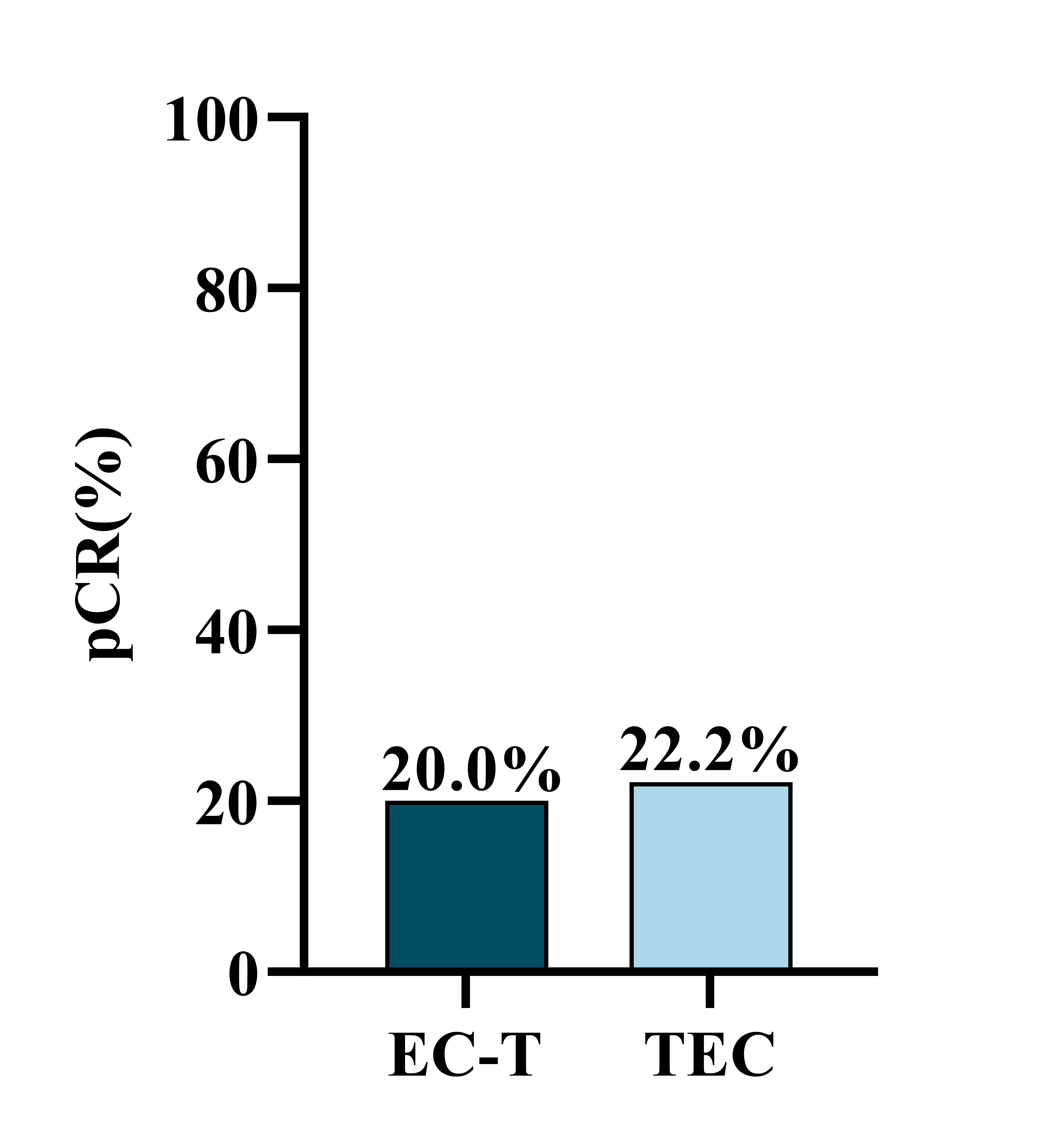


**Fig. S1.** Comparison of pCR rates for EC-T and TEC group. χ^2^ = 0.038.


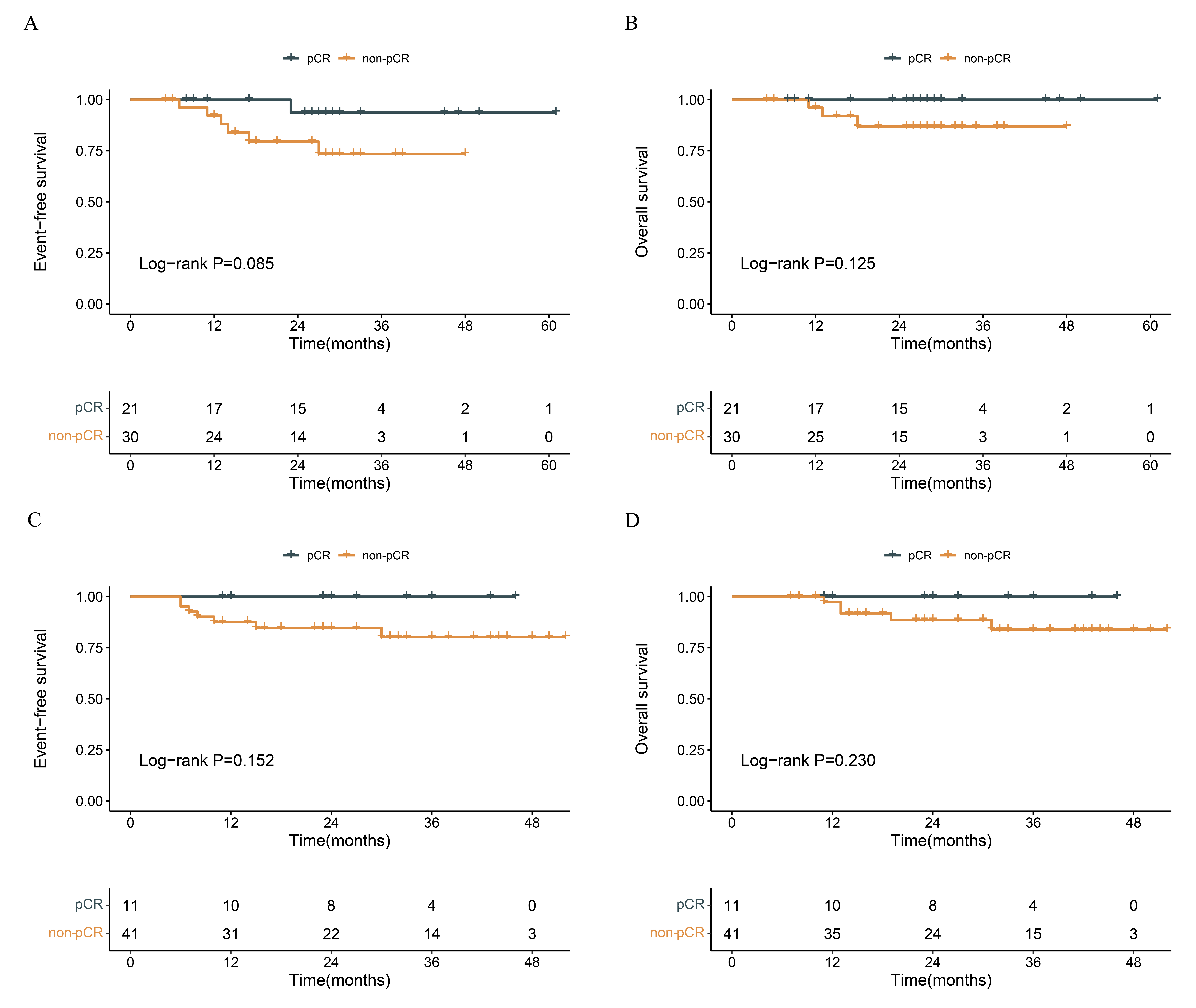


**Fig. S2.** Comparison of EFS and OS between pCR group with non-pCR group for Arm A and B. EFS (A) and OS (B) were compared between pCR group and non-pCR group for Arm A; EFS (C) and OS (D) were compared between pCR group and non-pCR group for Arm B.
